# Common Atrium: A Rare Cause of Acute Decompensated Heart Failure

**DOI:** 10.1155/2015/497891

**Published:** 2015-02-16

**Authors:** K. M. Karaye, S. A. Balarabe, M. M. Yakasai, I. M. Suleiman, H. Saidu, Aimé Bonny

**Affiliations:** ^1^Cardia Heart Clinic, 6/7 Hadejia Road, Kano, Nigeria; ^2^Department of Medicine, Bayero University/Aminu Kano Teaching Hospital, 3 New Hospital Road, Kano, Nigeria; ^3^Douala Cardiovascular Research Institute, Faculty of Medicine, University of Douala, Douala, Cameroon

## Abstract

We report a rare case of common atrium and acute decompensated heart failure most likely precipitated by acute bacterial pericarditis leading to premature death, in a 25-year-old male footballer. The silent course of the disease for decades as well as the diagnostic and management pitfalls of this case illustrates the importance of early detection by echocardiography and urgent appropriate treatment in intensive care settings to limit the poor prognosis of the condition.

## 1. Introduction

Common atrium (CA) is a rare form of atrioventricular septal defect characterized by lack of atrial septal tissue and attachment of mitral and tricuspid valves to the interventricular septum in the same anatomic plane [1]. A cleft mitral leaflet is almost always present with this condition [1]. Although CA occurs as an isolated congenital cardiac anomaly, it might also occur as part of Ellis van Creveld Syndrome, which is associated with skeletal and congenital heart malformations [2]. To the best of our knowledge, CA has not been reported as a cause of acute decompensated heart failure (ADHF). Here, we present a case of isolated common atrium presenting in ADHF.

## 2. Case Report

Mr. AD was a 25-year-old student who was an active footballer until ten days prior to presentation to Cardia Heart Clinic (CHC), Kano, Nigeria, when he started having dyspnoea on exertion, which progressed to dyspnoea at rest, associated with orthopnoea, paroxysmal nocturnal dyspnoea, easy fatigability, abdominal and leg oedema, upper right abdominal discomfort, easy satiety, and subsequently cough with frothy sputum. He denied having urinary symptoms, fever, or other symptoms. Prior to his self-referral to CHF, he had visited another clinic where a chest X-ray, complete blood count, and renal function assessment were carried out.

Physical examination revealed a young man in mild respiratory distress. There were no skeletal anomalies or digital clubbing, and he was 1.76 meters tall with body weight of 68 Kg. His axillary body temperature was 37.1°C, oxygen saturation on room air was 91%, and he had bilateral pitting pedal oedema extending to the shins. He had regular heart rate of 104 beats/minute, blood pressure of 100/80 mmHg, raised jugular venous pressure (approximately 8 cm), displaced apex, third heart sound with loud component of second heart sound, and grade IV mitral regurgitation and grade III tricuspid regurgitation murmurs. There were bilateral basal crepitations, soft and tender hepatomegaly of 12 cm below the right costal margin, and mild ascites. Other aspects of physical examination were not remarkable.

**Figure 1:**
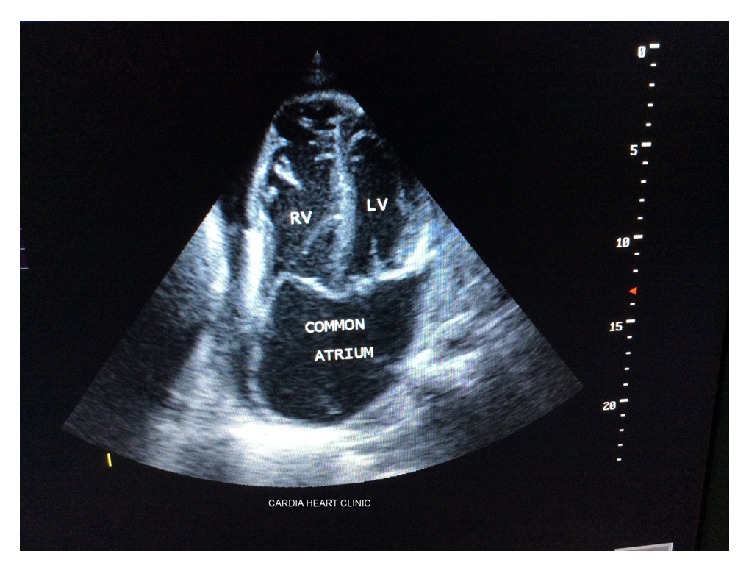
Two-dimensional echocardiogram apical 4-chamber view of the common atrium.

**Figure 2:**
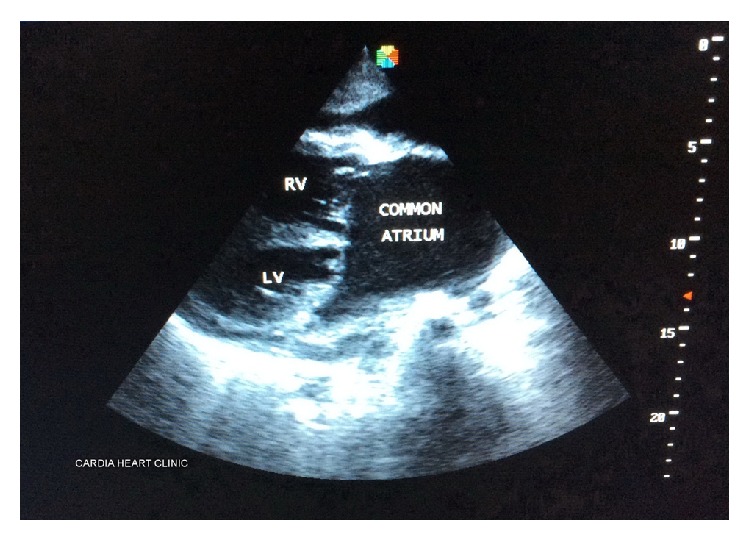
Two-dimensional echocardiogram parasternal long-axis view of the common atrium.

Chest X-ray revealed cardiomegaly, pulmonary venous congestion, and minimal left pleural effusion. Electrocardiogram showed sinus tachycardia with bifascicular block (complete right bundle branch block plus left posterior hemiblock). Echocardiogram (see Figures 1 and 2) revealed absent interatrial septum (common atrium) with deformed mitral and tricuspid valves (MV and TV, resp.), which were severely regurgitant. The right ventricle was dilated with a basal diameter of 50 mm, but left ventricular (LV) end diastolic diameter was normal (39 mm), and LV was hypercontractile (LV ejection fraction (LVEF) = 90%). His mean pulmonary artery systolic pressure was approximately 36.8 mmHg, consistent with moderate pulmonary hypertension. The pulmonary veins and superior vena were normally positioned. The pericardium was mildly thickened with moderate pericardial effusion (average echo free space = 15.5 mm). Fibrin strands were not seen within the effusion. The complete blood count showed total white blood cell count of 11.6 × 10^9^/L, neutrophils = 8.3 × 10^9^/L, lymphocytes = 2.6 × 10^9^/L, haemoglobin = 16.3 g/dL, haematocrit = 54.9%, and platelets = 522 × 10^9^/L. Serum creatinine was 92 *μ*mol/L, and other renal function parameters were all normal.

The overall diagnosis was ADHF with preserved LVEF and progressive secondary pulmonary hypertension, caused by CA, associated with moderate pericardial effusion and indirect evidence suggestive of acute bacterial infection.

After the initial cardiac evaluation at CHC which is an out-patient facility, the patient was immediately referred to the emergency unit of a tertiary level hospital in the city for admission and further treatment. The patient gave a written informed consent for his case to be reported. Unfortunately, the patient died about 20 hours after admission at the tertiary level hospital, but details of the in-patient management are not available to us, and postmortem examination was not carried out.

## 3. Discussion

This is the case of a young man who was an active footballer and who developed HF symptoms and deteriorated rapidly over the course of ten days. The case perhaps illustrates the natural course of CA, which the patient could tolerate well for more than two decades until an intervening cardiac event occurred. It also illustrates the indispensable role that echocardiography plays in the early management of HF.

The echocardiogram findings of absent interatrial septum, preserved LV geometry and LVEF, deformed MV and TV, significant MR and TR, and moderate pulmonary hypertension, as well as the ECG findings of bifascicular block, are all consistent with the diagnosis of CA. Absence of skeletal malformations has ruled out Ellis van Creveld Syndrome [2]. The ten-day course of his symptoms, absence of fibrin strands in the pericardial effusion, and absolute neutrophilia are all against a possible diagnosis of tuberculous pericarditis, but in support of acute bacterial pericarditis.

The rapidity with which the patient died strongly suggests that an acute event occurred at the tertiary level hospital which could have been pericardial tamponade, arrhythmias, acute pulmonary oedema, or pulmonary embolism. Management in the Intensive Care Unit and pericardiocentesis for therapeutic and diagnostic purposes were part of our recommendations, but it is not clear if they were timely carried out.

The present case adds to the long list of causes of HF with preserved ejection fraction (HFpEF) and ADHF and illustrates the associated high mortality [3]. Based on the clinical features alone and the background of the patient, the most likely differential diagnoses that most physicians would consider might have to be rheumatic mitral regurgitation, acute myocarditis, pericardial effusion, and dilated cardiomyopathy. Congenital heart disease would not have come up high in the list because of the silent course of the disease for over two decades. With echocardiography, however, the diagnosis was made and patient was immediately referred for further treatment. Therefore, HFpEF is indeed a heterogeneous condition with a diverse variety of causes and presentations, which partly explains why specific treatment is still unavailable.

## 4. Conclusion

We report a rare case of common atrium and acute decompensated heart failure most likely precipitated by acute bacterial pericarditis leading to premature death, in a 25-year-old male footballer. The silent course of the disease for decades as well as the diagnostic and management pitfalls of this case illustrates the importance of early detection by echocardiography and urgent appropriate treatment in intensive care settings to limit the poor prognosis of the condition.
